# Sexually Transmitted Diseases: Diagnosis and Control

**DOI:** 10.3390/ijerph19095293

**Published:** 2022-04-27

**Authors:** Teresa Fasciana, Giuseppina Capra, Dario Lipari, Alberto Firenze, Anna Giammanco

**Affiliations:** Department of Health Promotion, Mother and Child Care, Internal Medicine and Medical Specialties, University of Palermo, 90127 Palermo, Italy; giuseppina.capra@unipa.it (G.C.); dariolipari79@gmail.com (D.L.); alberto.firenze@unipa.it (A.F.); anna.giammanco@unipa.it (A.G.)

Sexually transmitted diseases (STDs)—or sexually transmitted infections (STIs)—are generally acquired through sexual contact. The bacteria, viruses or parasites involved are usually transmitted through sexual contact, including through bodily fluids or skin contact via vaginal, oral, and anal sex. STDs are a major health problem and mostly affect young people, not only in developing, but also in developed countries. The burden of disease that STIs represent globally is unknown for several reasons. Firstly, asymptomatic infections are common in many STIs; secondly, diagnostic methods are not available in some of the worst-affected countries; finally, surveillance systems are inexistent or very deficient in many areas of the world.

Accurate diagnostic tests for STIs are widely used in high-income countries. These are especially useful for the diagnosis of asymptomatic infections. However, diagnostic tests are largely unavailable in low- and middle-income countries. Where testing is available, it is often expensive and geographically inaccessible, and patients often need to wait a long time (or need to return) to receive results. As a result, follow-up can be impeded, and care or treatment can be incomplete.

For these reasons, several rapid tests for other STIs are under development and have the potential to improve STI diagnosis and treatment, especially in resource-limited settings.

This Special Issue includes full research articles and reviews focused on the epidemiology of STDs and on the specificity and sensibility of diagnostic tests used in the laboratory.

Among contributions reporting epidemiology data, Fasciana and coauthors reported a retrospective study that describes the sociodemographic profile and main sexual behaviors of patients attending a hospital in Palermo (Sicily, Italy) as predictors of STI risk. Patients were divided into subgroups: men who have sex with men (MSM), men who have sex with women (MSW), bisexual men, and females. Data were obtained through an anonymous questionnaire. Patients were tested for *Chlamydia trachomatis*, syphilis, *Mycoplasma genitalium* infection, genital herpes, and HPV infection. The data obtained showed that the most common profile of patients attending the clinic was that of an adult, Italian man with a high level of education, poor use of condoms, and a high number of partners. MSM had the highest sex-behavior-related risk for STIs. In addition, their results suggest that, in their geographic area, all STD teams need to implement counselling and recommendations to share with patients, as well as tips on how to approach sexual health education/counselling, thereby promoting patient-centered approaches and educational programs [1].

Haag et al. conducted a systematic literature search to determine *Chlamydia trachomatis* prevalence rates, identify risk groups, and delineate chlamydia-testing strategies. Their results suggest the use of a counselling as an opportunity to recommend chlamydia testing. Future approaches to identify target risk groups might include chlamydia prevalence testing among emergency contraception users attending community pharmacies. Furthermore, to reinforce the pharmacist’s new role as a gateway to health care, further pharmacy-based services might be developed to target individuals at particular risk for chlamydia infections in Switzerland, such as individuals with multiple infections including HIV or hepatitis C [2].

A prospective observational cohort study reported by Wang and coworkers was conducted among a group of men who have sex with men (MSM) who obtained pre-exposure prophylaxis (PrEP) in private clinics in Thailand and used it in Hong Kong. Participants completed two web-based self-administered surveys when obtaining PrEP in Thailand and three months afterwards. The results obtained showed that participants who perceived that they had a higher chance of STI infection (adjusted odds ratios (AOR): 1.90, 95% CI: 1.00, 3.75) and reported a higher intention to take up STI testing at baseline (AOR: 1.62, 95% CI: 1.05, 2.50) were more likely to receive STI testing during the follow-up period. Baseline perceptions that service providers would think they were engaging in risky behaviors because of PrEP use was negatively associated with the dependent variable (AOR: 0.51, 95% CI: 0.31, 0.86). Then, the need for service planning and health promotion related to STI testing for MSM “PrEP tourists” was underlined by the authors. Finally, the two papers recommended the implementation of specific programs of information and education [3]. 

Another study using anonymous self-administered questionnaires was conducted by Yamanaka and Kawata. In the paper, the authors report the characteristics of mother–daughter relationships among Japanese female university students and their associations with students’ sexual risk awareness. One of the main findings of this study is that sexual literacy and education about self-management may help to develop close communication between the mother and daughter and help these relationships in terms of trust and intimacy. These findings suggest that, as a specialized profession, we need to impress on society the importance of a good mother–daughter relationship so that young women can develop self-management abilities to help them avoid sexual risks [4].

In their results, Chan et al. showed that implementing HIV self-testing (HIVST) online is helpful in increasing HIV testing coverage among men who have sex with men (MSM) in Hong Kong. In this study, it was observed that about 80% of HIVST-online ever-users received some form of HIV testing during the study period, and most of them used HIVST-online again. Therefore, the implementation of HIVST-online has good potential to increase the regular HIV testing rate, which is very low among Chinese MSM. Regular HIV testing is important for MSM, as their sexual risk behaviors are continuous [5].

In the review entitled “Current and Future Trends in the Laboratory Diagnosis of Sexually Transmitted Infections”, Caruso and coauthors provide an updated overview of the current laboratory diagnostic tools used for these infections, highlighting their advantages, limitations, and point-of-care (POC) adaptability. The diagnostic applicability of the latest molecular and biochemical approaches was also reported. In the review, advances that have been made in the POC testing for STIs are reported, and more tests are in the pipeline. However, the need remains for the better integration of STI POC testing into healthcare systems. Due to the hidden nature of STIs, ensuring the extensive and rapid screening of at-risk people and their partners is pivotal to successfully control these infections [6].

In conclusion, this Special Issue collects papers with a unique aim: to improve the strategies used to increase screening and testing for STIs. This should help to better assess people’s risk of contracting an STIs and help people with STIs access treatment, improving their health and making it less likely that STIs will be spread to others.
